# Supplementary material for: Evaluation of the therapeutic efficacy of different doses of LT4 in pregnant women with high-normal TSH levels and TPOAb positivity in the first half of pregnancy
Source: Lipids Health Dis. 2024 Apr 10;23:101. doi: 10.1186/s12944-024-02099-9 (PMC11005176; doi:10.1186/s12944-024-02099-9)
Supplement: Supplementary file 1 — Supplementary Material 1 [file 12944_2024_2099_MOESM1_ESM.pdf]

AJE®

Editing Certificate

This document certifies that the manuscript

Evaluation of the Therapeutic Efficacy of Different Doses of LT4 in Pregnant Women with High-Normal TSH Levels and TPOAb Positivity in the First Half of Pregnancy

prepared by the authors

Xin Tian, Yajuan Xu, Yanjie Ban, Jingjing Li, Lin Hu, Dong Liu, Lulu Hu, Zongzong Sun, Miao Zhang, Chenchen Zhang, Yixin Wang, Pengkun Lin

was edited for proper English language, grammar, punctuation, spelling, and overall style by one or more of the highly qualified native English speaking editors at AJE.

This certificate was issued on **December 7, 2023** and may be verified on the AJE website using the verification code **1260-C9E5-83AC-4E2B-AE1F** .

AJE EDITING CERTIFIED

Neither the research content nor the authors' intentions were altered in any way during the editing process. Documents receiving this certification should be English-ready for publication; however, the author has the ability to accept or reject our suggestions and changes. To verify the final AJE edited version, please visit our verification page at aje.com/certificate. If you have any questions or concerns about this edited document, please contact AJE at support@aje.com.

AJE provides a range of editing, translation, and manuscript services for researchers and publishers around the world.
For more information about our company, services, and partner discounts, please visit aje.com.
