# Supplementary material for: Evaluation of the therapeutic efficacy of different doses of LT4 in pregnant women with high-normal TSH levels and TPOAb positivity in the first half of pregnancy
Source: Lipids Health Dis. 2024 Apr 10;23:101. doi: 10.1186/s12944-024-02099-9 (PMC11005176; doi:10.1186/s12944-024-02099-9)
Supplement: Supplementary file 2 — Supplementary Material 2 [file 12944_2024_2099_MOESM2_ESM.pdf]

PAPER NAME

2-5-Evaluation of the Therapeutic Efficacy of Different Doses of LT4 in Pregnant Women with High-Normal TSH Levels and TPOAb Positivity in the First Half of Pregnancy

AUTHOR

-

WORD COUNT

6527 Words

CHARACTER COUNT

38273 Characters

PAGE COUNT

22 Pages

FILE SIZE

154.7KB

SUBMISSION DATE

Mar 31, 2024 12:53 AM GMT+8

REPORT DATE

Mar 31, 2024 12:54 AM GMT+8

9% Overall Similarity

The combined total of all matches, including overlapping sources, for each database.

- 8% Internet database
- 7% Publications database
- Crossref database
- Crossref Posted Content database

Excluded from Similarity Report

- Bibliographic material
- Manually excluded text blocks

RESEARCH ARTICLE

Evaluation of the Therapeutic Efficacy of Different Doses of LT4 in Pregnant Women with High-Normal TSH Levels and TPOAb Positivity in the First Half of Pregnancy

Xin Tian,[1] Yajuan Xu,[1*] Yanjie Ban,[1] Jingjing Li,[1] Lin Hu,[1] Dong Liu,[1] Lulu Hu,[1] Zongzong Sun,[1] Miao Zhang,[1] Chenchen Zhang,[1] Yixin Wang,[1] Pengkun Lin[1]

[1]Department of Obstetrics and Gynecology, The Third Affiliated Hospital of Zhengzhou University, Zhengzhou, Henan, China;

Correspondence: cnzzzsl@163.com

Abstract

Background: The objective was to investigate the efficacy of different doses of levothyroxine therapy among pregnant women exhibiting high-normal thyroid stimulating hormone levels and positive thyroid peroxidase antibodies throughout the first half of pregnancy.

Methods: Pregnant women exhibiting high-normal thyroid stimulating hormone levels and thyroid peroxidase antibodies positivity throughout the initial half of pregnancy were selected from January 2021 to September 2023. Based on the different doses of levothyroxine, the pregnant women were categorized into the nonintervention group (G_0, 122 women), 25 μg levothyroxine intervention group (G_{25}, 69 women), and 50 μg levothyroxine intervention group (G_{50}, 58 women). Serum parameters, gastrointestinal

symptoms, small intestinal bacterial overgrowth (SIBO), maternal and neonatal outcomes were compared after the intervention among the three groups.

Results: After the intervention, in the G_{25} and G_{50} groups, the thyroid stimulating hormone, triglyceride and low-density lipoprotein levels were notably less in contrast to those in the G_0 group ($P < 0.05$). The rates of abdominal distension and SIBO in the G_{25} and G_{50} groups were notably lower in contrast to the G_0 group ($P = 0.043$ and 0.040, respectively). The G_{50} group had a lower rate of spontaneous abortion and premature membrane rupture than the G_0 group ($P = 0.01$ and 0.015, respectively). Before 11^{+2} weeks of gestation and at thyroid peroxidase antibodies levels ≥ 117 IU/mL, in contrast to the G_0 group, the G_{50} group experienced a decreased rate of spontaneous abortion ($P = 0.008$). The G_{50} group had significantly higher newborn weight than the G_0 group ($P = 0.014$), as well as a notably longer newborn length than the G_0 and G_{25} groups ($P = 0.005$).

Conclusions: Levothyroxine can improve blood lipid levels, gastrointestinal symptoms and reduce the occurrence of SIBO, spontaneous abortion and premature rupture of membranes in the gravid females with high-normal thyroid stimulating hormone levels and thyroid peroxidase antibodies positivity during the first half of pregnancy, with 50 μg levothyroxine has better efficacy.

Keywords: levothyroxine, thyroid stimulating hormone, thyroid peroxidase antibodies, lipid, SIBO, pregnancy outcome

Introduction

With the advancement of medicine, thyroid dysfunction has gradually become a focal point of attention for clinicians, particularly high-normal thyroid stimulating hormone (TSH) levels and thyroid peroxidase antibody (TPOAb) positivity, which are among the key concerns for obstetricians and gynecologists. High-normal TSH refers to TSH levels ranging from 2.5 mIU/L to the uppermost extent of normal, accompanied by Free thyroxine (FT4) within the normal parameters[1]. TPOAb positivity refers to levels of thyroid peroxidase antibodies (TPOAb) exceeding the maximum value indicated by the assay's reference range,

with an incidence rate of 5%-14% in pregnant women[2]. Current evidence suggests that a high-normal TSH level with TPOAb positivity elevates the likelihood of unfavorable maternal-fetal outcomes, for instance spontaneous miscarriage, preterm delivery, gestational hypertension, as well as fetal intrauterine distress[3, 4].

Karbownik-Lewińska et al. observed that individuals with high-normal TSH levels are prone to abnormal triglycerides (TG)[5]. Disrupted lipid levels create a lipotoxic environment, characterized by oxidative stress, inflammation, and alterations in lipid transport and metabolism, ultimately diminishing trophoblast invasion further. This scenario affects placental metabolism, function, and fetal development[6]. Studies conducted by Rohlfing and Chen further emphasized the association of elevated TG with total cholesterol (TC) levels during pregnancy, highlighting a heightened risk of complications, including gestational diabetes mellitus (GDM), macrosomia, and preterm birth[7, 8]. Moreover, gut microbiota has gradually attracted attention in recent years. Small intestinal bacterial overgrowth (SIBO), reflecting disruptions in intestinal flora, is characterized by abnormal bacterial proliferation in the small intestine, leading to gastrointestinal symptoms[9]. SIBO not only triggers gastrointestinal symptoms such as abdominal distension and constipation in patients, but also induces metabolic disturbances, disrupts intestinal immunity, leads to intestinal permeability, and consequently contributes to immune system dysregulation[10]. The compromised immune tolerance in gravid females heightens the likelihood of unfavorable maternal-fetal outcomes, for instance spontaneous abortion[11].

Guidelines for prevention and management of thyroid diseases during pregnancy and perinatal period in 2022 recommended levothyroxine (LT4) replacement therapy for gravid females exhibiting the normal high range of TSH levels and TPOAb positivity[1]. However, international guidelines do not distinctly advocate or discourage LT4 therapy for gravid females with the normal high range of TSH levels and TPOAb positivity[12]. Moreover, there is a dearth of research on the dosage of LT4 treatment for this specific population. Therefore, this study aimed to delve into the optimal dosage of LT4 for pregnant women with the normal high range of TSH levels and TPOAb positivity to guide clinical work.

Materials and methods

Study cohort

From January 2021 to September 2023, the study retrospectively investigated pregnant women undergoing thyroid function tests at the Third Affiliated Hospital of Zhengzhou University. In all, 249 pregnant women were included, comprising the untreated group (G_0, n=122), the group treated with 25 µg LT4 (G_{25}, n=69), and the group treated with 50 µg LT4 (G_{50}, n=58).

The criteria for inclusion were as outlined below: 1) gestational age should not exceed 20 weeks; and 2) thyroid function within the reference ranges operated by the Clinical Laboratory at Zhengzhou University Third Affiliated Hospital: a TPOAb level > 34 IU/mL; an FT4 level of 12.1 pmol/L - 19.6 pmol/L as well as levels of TSH ranging from 2.5 mIU/L to 4.0 mIU/L during initial period of pregnancy; and an FT4 level of 9.63 pmol/L - 17.0 pmol/L, and levels of TSH ranging from 2.5 mIU/L to 4.1 mIU/L in mid-pregnancy.

The criteria for exclusion were as outlined below: 1) concurrent hypothyroidism, subclinical hypothyroidism, or hyperthyroidism; 2) assisted reproduction; 3) multiple pregnancy; 4) coexisting autoimmune diseases such as antiphospholipid syndrome, thrombophilia, and others; 5) severe gastrointestinal diseases, a history of gastrointestinal surgery, or the use of medications affecting the gut microbiota, such as probiotics and prebiotics; 6) irregular prenatal care, the inability to track medication usage and the lack of follow-up thyroid function tests; and 7) adverse reactions to LT4 treatment.

Data extraction

For participants included in the study, data on personal attributes [age, body mass index (BMI)], reproductive background, past pregnancies, thyroid function levels at the onset of thyroid dysfunction and after 4 weeks of LT4 (Merck Health KgaA, registration number: H20140052) treatment, serum indicators such as total cholesterol, triglycerides, high-density lipoprotein (HDL), and low-density lipoprotein (LDL) concentrations, digestive symptoms (diarrhea, bloating, constipation), SIBO, and LT4 dosage were recorded. Probiotics (Sienkang, National drug approval: S20060010) and prebiotics (Risikon®, production

license number: SC13061011200721) were administered to pregnant women with SIBO. Patient records were reviewed for antenatal conditions (premature rupture of membranes, fetal intrauterine distress, placental abruption, etc.), perinatal outcomes (emergency cesarean section, amount of bleeding during delivery, etc.), and neonatal outcomes (weight, length, Apgar score, neonatal admission rate, etc.).

Thyroid function laboratory assays

Including TSH, FT4, and TPOAb, thyroid function parameters were determined in the laboratory using electrochemical luminescence immunoassay technique (Cobas e 801, Roche Diagnostics, Mannheim, Germany). The precise ranges of values used as a reference for assessing thyroid gland function, as operated by the Clinical Laboratory in conformity with the "Guidelines for prevention and management of thyroid diseases during pregnancy and perinatal period", are as follows: TPOAb: 0 IU/ml - 34 IU/ml. In early pregnancy, FT4 is considered normal within the range of 12.1 pmol/L - 19.6 pmol/L, and TSH is considered normal within the range of 0.33 mIU/L - 4.0 mIU/L. In the midst of pregnancy, the FT4 range is 9.63 pmol/L - 17.0 pmol/L, and that for TSH is 0.45 pmol/L - 4.1 pmol/L.

Determining small intestinal bacterial overgrowth

Diagnostic criteria were defined using the standards of Breath Tracker SC (QuinTron Instruments, Milwaukee, WI, USA) in accordance with the North American consensus: 1) An initial rise of ⩾20 ppm in hydrogen in the first 90 minutes after substrate ingestion indicated an SIBO diagnosis; 2) A methane level equal to or exceeding 10 ppm at any juncture was deemed indicative of SIBO; 3) if the abundances of methane and hydrogen fail to meet the above values and their combined sum surpasses the baseline sum, accompanied by methane abundance exceeding 15 ppm within 90 minutes, a diagnosis of SIBO is considered[13]. Due to the shorter blind time in the Asian population compared to Western populations and the fact that lactulose shortens the blind time, this study adopted a 20-minute interval for breath sampling to reduce the false-positive rate[14].

Pregnancy outcomes

Pregnancy-related outcomes encompassed maternal complications, comorbidities, and neonatal outcomes. Maternal complications and comorbidities included spontaneous abortion, preterm birth, GDM, gestational hypertension, placental abruption, fetal intrauterine distress, premature rupture of membranes, emergency cesarean section, and intrapartum hemorrhage. Neonatal outcomes comprised newborn weight, length, Apgar scores, and neonatal admission rate.

Statistical analysis

Using SPSS 26.0 (SPSS, Chicago, IL, USA), the numerical evaluations were conducted. Normality tests were performed for continuous variable data, and continuous variables following a normal distribution are presented using the mean along with the standard deviation. To compare variations across multiple groups, a post hoc comparison was conducted following the utilization of Analysis of variance (ANOVA). Nonnormally distributed continuous variables are represented by medians and quartiles, and rank sum test was employed for intergroup comparisons, along with multiple analyses. Percentage and frequency distributions were used to present categorical data, with comparisons made through either the chi-square test or Fisher's exact probabilistic test. In this study, the significance level of $P < 0.05$ was utilized.

Results

Basic clinical characteristics of the included pregnant women

The fundamental details of the pregnant subjects, including age, BMI, gestational weeks, adverse obstetric history, and other features (Table 1). In comparing the three groups, no statistically significant differences emerged with respect to age, BMI, gestational weeks, adverse obstetric history, parity, and gravidity. In terms of serum indicators, the three groups exhibited similarity in thyroid function markers such as FT4, TSH, and TPOAb, as well as in lipid levels including TC, TG, HDL, and LDL, with no statistically significant differences observed.

Table 1 General information comparison among pregnant women

Parameter	G_0 (n=122)	G_{25} (n=69)	G_{50} (n=58)	*P*
Age (years)	30.00(27.00-33.00)	31.00(29.00-34.00)	31.00(28.00-32.00)	0.250
BMI (kg/m^2)	21.39(19.36-23.49)	21.88(20.32-23.78)	21.64(20.21-23.55)	0.308
Gestational weeks	11.65(7.40-13.50)	8.57(6.43-12.43)	11.79(7.40-13.47)	0.084
Adverse obstetric history	0(0-0)	0(0-1)	0(0-0)	0.059
Pregnancy	2(1-2)	2(1-3)	2(1-2)	0.355
Parity	0(0-1)	0(0-1)	0(0-1)	0.920
FT4 (pmol/L)	15.00(13.90-16.43)	15.10(13.60-16.80)	14.85(13.20-16.83)	0.529
TSH (mIU/L)	3.02(2.77-3.41)	3.04(2.80-3.51)	3.17(2.85-3.67)	0.171
TPOAb (IU/mL)	125.00(72.08-251.25)	169.00(85.85-294.50)	150.00(85.45-279.25)	0.184
TC (mmol/L)	4.63±0.68	4.73±0.50	4.56±0.54	0.288
TG (mmol/L)	1.34±0.41	1.44±0.49	1.37±0.33	0.263
HDL (mmol/L)	1.75±0.34	1.82±0.40	1.71±0.37	0.199
LDL (mmol/L)	2.53±0.63	2.68±0.55	2.52±0.69	0.243

Numerical data are exhibited as either the median along with its interquartile range or as the mean along with the standard deviation. All comparisons were corrected using Bonferroni correction.

Serum parameters in the three groups of pregnant women receiving different doses of LT4 treatment

Following the intervention, compared to those in the G_0 group, the FT4 levels from the G_{25} and G_{50} groups were conspicuously raised ($P < 0.001$), Similarly, the TSH levels in the G_{25} and G_{50} groups were notably decreased compared to G_0 group ($P < 0.001$) (Table 2). Regarding blood lipids, TG and LDL levels in the

G_{25} and G_{50} groups were notably decreased from the G_0 group (P = 0.046 and P = 0.033, respectively). Nevertheless, there were no notable variances in TC and HDL levels among the three groups.

Table 2 Serum comparisons in the three groups of pregnant women receiving different doses of LT4 treatment

Parameter	G_0 (n=122)	G_{25} (n=69)	G_{50} (n=58)	P
FT4 (pmol/L)	12.75(11.50-13.70)	13.70(12.10-14.90) a	14.05(12.68-16.13) b	< 0.001
TSH (mIU/L)	3.66(2.45-4.16)	2.37(1.77-2.89) c	2.07(1.55-2.98) d	< 0.001
TPOAb (IU/mL)	67.55(37.60-140.25)	82.80(46.80-179.00)	99.25(54.53-200.53)	0.072
TC (mmol/L)	5.91±0.90	5.87±0.82	5.67±0.69	0.210
TG (mmol/L)	3.26±0.94	3.01±0.75	2.97±0.79	0.046
HDL (mmol/L)	2.17±0.44	2.04±0.49	2.08±0.47	0.148
LDL (mmol/L)	4.23±0.81	4.02±0.79	3.93±0.67 e	0.033

Numerical data are exhibited as either the median along with its interquartile range or as the mean along with the standard deviation. a demonstrates P <0.01 in contrast to the G_0 group; b, c, and d indicate P <0.001 in contrast to the G_0 group; e indicates P < 0.05 in comparison to the G_0 group. All comparisons were corrected using Bonferroni correction.

Gastrointestinal symptoms and the SIBO rate in the three groups of pregnant women receiving different doses of LT4 treatment

Before the intervention, there were no statistically notable disparities in gastrointestinal symptoms (diarrhea, bloating, constipation) or the SIBO rates among the three groups. Following the LT4 intervention, the occurrence rates of bloating and SIBO in the G_{25} and G_{50} groups were of a lower value than those in the G_0 group, exhibiting a statistically significant difference (P = 0.043 and P = 0.04, respectively). Among the three groups, there were no notable variances in diarrhea and constipation rates (Table 3).

Table 3 Gastrointestinal symptoms and the SIBO rate comparisons among the three groups

Parameter	G_0 (n=122)	G_{25} (n=69)	G_{50} (n=58)	*P*
Diarrhea (%)	18.9(23/122)	11.6(8/69)	10.3(6/58)	0.217
Bloating (%)	28.7(35/122)	17.4(12/69)	13.8(8/58)	0.043
Constipation (%)	21.3(26/122)	11.6(8/69)	13.8(8/58)	0.176
SIBO+ (%)	32.0(39/122)	18.8(13/69)	17.2(10/58)	0.040

SIBO: Small Intestinal Bacterial Overgrowth.

Maternal outcomes in the three groups

According to the results shown in Table 4, the rates of spontaneous pregnancy loss and premature membrane rupture in the G_{50} group were notably lower as compared with the G_0 group (pregnancy loss showing a *P* value of 0.01, while membrane rupture exhibited a *P* value of 0.015). The incidence rates of preterm birth, GDM, gestational hypertension, placental abruption, fetal intrauterine distress, and emergency cesarean section, as well as intrapartum hemorrhage, decreased in the G_{50} group in contrast to those in the G_0 group, without any notable differences. Among the women who experienced spontaneous abortion in the G_0, G_{25}, and G_{50} groups, there were no cases of bleeding during delivery.

Table 4 Contrast of maternal outcomes across the three groups of pregnant women

Parameter	G_0 (n=122)	G_{25}(n=69)	G_{50} (n=58)	*P*	P_1	P_2	P_3
Spontaneous abortion (%)	17.(21/122)	13.0(9/69)	3.4(2/58)	0.036	0.447	0.01	0.055
Preterm birth (%)	6.6(8/122)	10.1(7/69)	1.7(1/58)	0.164	0.376	0.275	0.07
GDM (%)	23.0(28/122)	27.(19/69)	13.8(8/58)	0.168	0.48	0.151	0.059
Gestational hypertension (%)	5.7(7/122)	7.2(5/69)	0(0/58)	0.105	0.759	0.098	0.062
Placental abruption (%)	0.8(1/122)	1.4(1/69)	1.7(1/58)	0.8	1	0.542	1
Fetal intrauterine distress (%)	19.7(24/122)	11.6(8/69)	10.3(6/58)	0.162	0.151	0.117	0.823
Premature rupture of	18.9(23/122)	17.4(12/69)	5.2(3/58)	0.049	0.802	0.015	0.034

membranes (%)							
Emergency cesarean section (%)	11.5(14/122)	8.7(6/69)	5.2(3/58)	0.387	0.547	0.117	0.507
Intrapartum hemorrhage (ml)	280(200-305)	300(220-315)	230(180-330)	0.136	0.357	0.169	0.054

P_1 illustrates the G_0 and G_{25} groups comparison, P_2 illustrates the G_0 and G_{50} groups comparison, and P_3 illustrates the G_{25} and G_{50} groups comparison; All comparisons were corrected using Bonferroni correction.

Spontaneous abortion rates among pregnant women taking different doses of LT4 at different gestational weeks and TPOAb levels

The area under the curve (AUC) for gestational age corresponding to live birth was 0.682 (95% CI: 0.598-0.766, P = 0.001), with a Youden index of 0.396, according to receiver operating characteristic (ROC) analysis. The corresponding cutoff value was 11.22 weeks. At a cutoff of 11.22 weeks, the specificity for live birth during this period was 87.5%, and the sensitivity was 52.10%.

Table 5 shows the spontaneous abortion rates of pregnant women at different gestational weeks and TPOAb levels. There was a notable variance in the spontaneous abortion rates among the three groups (P = 0.036). The ROC curve cutoff for gestational weeks was 11.22, i.e., 11^{+2} weeks. In the subgroup analysis of spontaneous abortion rates, the G_{50} group had a notably lower spontaneous abortion rate than the G_0 group when the gestational age was before 11^{+2} weeks and the TPOAb level was ≥ 117 IU/mL (P = 0.018). There was no notable variation in the spontaneous pregnancy loss rates among the three groups when the gestational age was before 11^{+2} weeks and the TPOAb level was < 117 IU/mL or when the gestational age was between 11^{+2} weeks and 20 weeks.

Table 5 Comparison of spontaneous abortion rates among pregnant women at different gestational weeks and TPOAb levels

Parameter	TPOAb (IU/mL)	G_0 (n=122) (%)	G_{25} (n=69) (%)	G_{50} (n=58) (%)	*P*
Gestational weeks < 11.22	-	30.0(18/60)	20(9/45)	3.7(1/27)	0.021
	< 117	17.9(5/28)	18.8(3/16)	0(0/9)	0.526
	≥ 117	40.6(13/32)	20.7(6/29)	5.6(1/18) f	0.018
Gestational weeks ≥ 11.22	-	4.8(3/62)	0(0/24)	3.2(1/31)	0.810
	< 117	6.7(2/30)	0(0/5)	6.7(1/15)	1.0
	≥ 117	3.1(1/32)	0(0/19)	0(0/16)	1.0

f indicates $P = 0.008$ compared to the G_0 group. All comparisons were corrected with Bonferroni correction.

Neonatal outcomes among the three groups of pregnant women

In the G_{50} group, the weight of neonates was notably greater compared to the G_0 group ($P = 0.014$), and the length of neonates from the G_{50} group was notably longer than that of neonates from both the G_0 and G_{25} groups ($P = 0.005$). Nevertheless, there were insignificant differences in terms of the occurrence of macrosomia, Apgar scores at 1 and 5 minutes, or the rate of neonatal hospitalization among the three groups, as shown in Table 6. In the G_0 group, one neonate was transferred to the neonatal intensive care unit, with one case of severe asphyxia and one case of mild asphyxia. There was one case of the neonatal intensive care unit transfer in the G_{25} group.

Table 6 Neonatal outcomes comparisons in three pregnancy groups

Parameter	G_0 (n=122)	G_{25} (n=69)	G_{50} (n=58)	*P*	P_1	P_2	P_3
Macrosomia (%)	1.6(2/122)	4.3(3/69)	5.2(3/58)	0.36	0.354	0.33	1
Neonatal admission rate (%)	18.0(22/122)	18.8(13/69)	13.8(8/58)	0.719	0.91	0.462	0.446
Neonatal weight (g)	3240.0(2942.5-3455.0)	3260.0(2980.0-3475.0)	3350.0(3142.5-3565.0)	0.046	0.529	0.014	0.102
Length (cm)	51(50-52)	51(49-52)	51 (51-52.5)	0.005	0.965	0.001	0.014

Apgar 1min	10(10-10)	10(10-10)	10(10-10)	0.077	0.218	0.037	0.328
Apgar 5min	10(10-10)	10(10-10)	10(10-10)	0.185	0.823	0.061	0.089

P_1 refers to the comparison between the G_0 and G_{25} groups, P_2 refers to the comparison between the G_0 and G_{50} groups, and P_3 refers to the comparison between the G_{25} and G_{50} groups. All comparisons were corrected using Bonferroni correction.

Discussion

Thyroid hormones fulfill an essential function in fetal brain development, with fetal thyroid development occurring between 8-12 weeks and the thyroid becoming functional at approximately 18-20 weeks[15]. Consequently, during the first half of pregnancy (≤ 20 gestational weeks), fetal development significantly relies on maternal thyroid hormone provision. High-normal TSH levels and TPOAb positivity can lead to adverse pregnancy outcomes in pregnant women. Some studies suggest that reproductive-age women with the normal high range of TSH levels and TPOAb positivity should receive LT4 treatment[5, 16]. However, there is a lack of research on the appropriate dosage of LT4 for such patients, both domestically and internationally. Therefore, the primary objective of this research was to evaluate the therapeutic efficacy of different LT4 doses for gravid females with high-normal TSH levels and TPOAb positivity in the initial half of pregnancy.

This study found that the FT4 levels in the G_{25} and G_{50} groups were notably elevated compared to those in the G_0 group after LT4 intervention, while the TSH levels were significantly lower. Supplemented LT4 can be metabolized into FT4 in the body, which is subsequently transformed into triiodothyronine (T3) by the action of deiodinase. T3, through negative feedback regulation[17], reduces the production and release of thyrotropin-releasing hormone in the hypothalamus, subsequently decreasing the synthesis and secretion of TSH[18]. The TG and LDL

levels in the 50ug LT4 intervention group were clearly lower and significantly reduced compared to those in the non- intervention group. Michalopoulou et al. observed that individuals with high-normal TSH levels taking 50 μg LT4 could significantly reduce LDL levels compared with patients with low-normal TSH levels, which aligns with the outcomes observed in this research; however, they also found that it could reduce TC levels[19], which is contradictory to the results of this study. This may be caused by the incomplete consistency of the included populations. The mechanisms of improvement in blood lipids after taking LT4 in gravid females exhibiting high-normal TSH levels and TPOAb positivity in the first half of pregnancy may be as follows: 1) T3, converted from supplemented LT4, binds with thyroid hormone receptors (TRs)., forming a complex with transcriptional activation, which may activate adenosine monophosphate-activated protein kinase and increase the number of autophagosomes and lysosomes[20]; then, autophagosomes, by engulfing TG, fuse with lysosomes, promoting the degradation of TG[21]. 2)Supplemented LT4, after conversion to T3 in the body, reduces TSH levels through negative feedback regulation, decreasing the stimulation of steroid regulatory element-binding protein by TSH; subsequently, proprotein convertase subtilisin/kexin type 9 (PCSK9) in liver cells is reduced. Lowering PCSK9 levels can increase LDL receptors located on liver cells, promoting the degradation of LDL and thereby improving lipid metabolism[22, 23]. Therefore, this study suggests that gravid females with high-normal TSH levels and TPOAb positivity who take LT4 in the first half of pregnancy may improve their lipid metabolism.

The study also found that the occurrence of bloating and SIBO in the G_{50} group after the intervention showed a reduction compared to the G_0 group. Komiyama et al. discovered that administering LT4 to neonates with extremely low birth weight who suffer from hypothyroxinemia can improve abdominal distension symptoms[24], consistent with the research findings. Thyroid

hormones participate in various processes in the intestines, with the intestinal epithelium being one of the target sites for thyroid hormones. The possible mechanisms for improving gastrointestinal symptoms and SIBO positivity rate after taking LT4 in patients with the normal high range of TSH levels and TPOAb positivity in the first half of pregnancy include the following: 1) LT4 conversion to T3, which can bind to TRs in the intestinal epithelium, activate TRs binding to specific DNA, promote its expression, induce intestinal cell proliferation, maintain intestinal homeostasis, and alleviate bloating symptoms by reducing the gas production and fermentation caused by abnormal microbial communities[25, 26]. 2) The previous findings of our research group revealed an increased abundance of Roseburia, a hydrogen-producing bacterium, in pregnant women with thyroid dysfunction[27]. Supplemented LT4 enters the body and is convert into T3, which can bind to TRs in macrophages, promote anti-inflammatory response[28], reduce the occurrence of leaky gut, reduce hydrogen-producing bacteria and–methanogens, correct intestinal flora disorders, and reduce the rate of SIBO in pregnant women[29, 30]. Therefore, this study suggests that gravid females with the normal high range of TSH levels and TPOAb positivity in the first half of pregnancy who take LT4 may improve gastrointestinal symptoms and SIBO by enhancing the intestinal barrier and regulating the intestinal microenvironment.

The results of this research disclosed that the occurrence of spontaneous pregnancy loss and premature membrane rupture was less frequent in the G_{50} group in comparison to the G_0 group. The research conducted by Yang et al. demonstrated that the use of LT4 reduced the incidence of pregnancy loss in TPOAb positive gravid females[31], which aligns with findings of this study. However, the research conducted by Di Girolamo et al. suggested that LT4 did not significantly improve the likelihood of miscarriage in pregnant women positive for TPOAb[32], which contradicts the results. This discrepancy may be attributed to differences in inclusion criteria,

regional variations, and long intervals between studies. The mechanisms of improving spontaneous abortion and premature rupture of membranes after taking LT4 in gravid females with high-normal TSH levels and TPOAb positivity in the first half of pregnancy include the following: 1) LT4 supplementation can reduce TG, decreasing the production of fatty acids by TG degradation. This, in turn, mitigates the disruption of T-cell subset balance by fatty acids[33], reducing systemic inflammation[34]. Additionally, LT4 supplementation may restore intestinal microbiota homeostasis, reducing inflammation caused by intestinal permeability and thereby restoring immune accommodation at the interface between mother and fetus, ultimately lowering the risk of miscarriage[35, 36]. 2) After supplementing LT4, the levels of thyroxine (T4) and T3 increased in the body, T4 and T3 can increase the expression of oncofetal fibronectin and integrin α5β1, facilitating adhesion between extravillous trophoblast and the decidua. This upregulates the expression of metalloproteinases, enhancing the degradation of the endometrial extracellular matrix. These actions contribute to the remodeling of spiral arteries and the generation of decidual blood vessels by increasing the presence of factors like angiogenesis promoting factor and trophoblastic growth stimulant, ultimately reducing the risk of miscarriage[37]. 3) LT4, when converted by deiodinases into active T3, can bind to TRs in decidual cells, promoting the transcription of genes involved in thyroid hormone responses. This process increases the expression of nitric oxide synthase 2 (NOS2) and the anti-inflammatory cytokine interleukin-10 (IL-10) in the decidua, preventing inflammation-induced weakening and rupture of the amniotic sac, and consequently reducing the risk of premature rupture of the amniotic sac[37, 38]. Therefore, this study suggests that the administration of 50 μg LT4 to gravid females with the normal high range of TSH levels and TPOAb positivity in the first half of pregnancy may be more

effective in improving harmful obstetric outcomes for instance miscarriage and premature rupture of membranes.

The investigation further explored the impact of different doses of LT4 on spontaneous abortion in gravid females at various gestational weeks and with different TPOAb levels. ROC analysis yielded a gestational week cutoff value of 11 weeks and 2 days. Before 11^{+2} weeks of gestation, at TPOAb levels $\geq$ 117 IU/mL, the G_{50} group exhibited a notably lower rate of spontaneous abortion in comparison to the G_0 group. The study results align with those reported in prior research indicating that LT4 supplementation in women with high-normal TSH levels during early pregnancy can reduce the incidence of spontaneous abortion[39]. For embryo implantation and pregnancy, decidualization is necessary in early pregnancy. In women with the normal high range of TSH levels and TPOAb levels $\geq$ 117 IU/mL before 11^{+2} weeks of gestation, the administration of 50 μg LT4 may reduce the risk of spontaneous abortion for the following reasons: 1) LT4 supplementation may reduce the risk of hypothyroidism caused by elevated TPOAb levels[36], and sufficient TH levels can promote the upregulation of progesterone receptor-AB mRNA induced by ovarian steroid hormones, facilitating progesterone receptor signal transduction, aiding endometrial decidualization, and promoting embryo implantation[40], thereby lowering the risk of spontaneous abortion. 2) T4 and T3, which are converted though supplementation with LT4, facilitate the secretion of human placental prolactin, estradiol, progesterone, and human chorionic gonadotropin as well as the expression of placental growth factors when they reach the placenta. This promotes the differentiation of the trophoblastic layer and the formation of decidual angiogenesis, facilitating normal placental development[37, 41], and reducing the risk of spontaneous abortion in pregnant women. Therefore, this study recommends supplementation with 50 μg LT4 for gravid females with high-normal TSH levels and TPOAb

levels $\geq$ 117 IU/mL identified before 11^{+2} weeks of gestation, as it can successfully decrease the risk of spontaneous abortion.

The study found that the weight of neonates from the G_{50} group notably exceeded that of neonates from the G_0 group, and the length of neonates from the G_{50} group was notably longer compared to that of neonates from the G_0 and G_{25} groups. In their study, Huget-Penner et al. mentioned that LT4 supplementation in gravid females with hypothyroidism can improve the pregnancy outcomes of low birth weight infants[42], which supports the outcomes observed in this study. This may be attributed to the supplementation of 50 μg LT4, allowing more thyroid hormones to pass through the placenta into the fetal circulation, modifying fetal gene expression, cell proliferation, and differentiation during organ formation, thereby promoting fetal development[43]. Therefore, this study recommends that in gravid females with the normal high range of TSH levels and TPOAb positivity in the initial half of pregnancy, 50 μg LT4 is more effective in supplementing thyroid hormones, and improving neonatal developmental status, while the effect of 25 μg LT4 is not significant.

Strengths and limitations

This study had some advantages. First, it used strict diagnostic criteria to evaluate the outcomes of gravid females with high-normal TSH levels and TPOAb positivity taking different doses of LT4. Secondly, it conducted a more detailed subgroup analysis of spontaneous abortion among pregnant women across different gestational ages and TPOAb levels to understand the differences between the subgroups. Some limitations were also evident in this study. Although this study tried its best to ensure the representativeness and reliability of the sample, the sample size was limited and there may be some biases. In order to overcome these limitations, future studies will expand the sample

size and strengthen follow-up, monitoring and quality control during the research process to reduce research bias.

Conclusions

There is currently a lack of research on the impact of doses of LT4 on the outcomes of pregnancy among women with high normal TSH values and TPOAb positive in the initial half of pregnancy. This study found that gravid females with the normal high range of TSH levels and TPOAb positivity in the first half of pregnancy who supplemented with 50 μg LT4 were more effective in improving their blood lipid status and gastrointestinal symptoms, reducing the incidence of SIBO and premature rupture of membranes, and before 11^{+2} weeks, TPOAb ⩾ 117IU/mL proved more beneficial in mitigating the risk of spontaneous abortion.

Abbreviations

TPOAb: Thyroid peroxidase antibody; TSH: Thyroid stimulating hormone; FT4: Free thyroxine; GDM: Gestational diabetes mellitus; TG: Triglyceride; TC: Total cholesterol: SIBO: Small intestinal bacterial overgrowth; LT4: Levothyroxine; HDL: High density lipoprotein; BMI: Body mass index;LDL: Low density lipoprotein; ROC: Receiver operating characteristics; NICU: Neonatal intensive care unit; T3: Triiodothyronine; T4: Thyroxine; TRs: Thyroid hormone receptors; PCSK9: Proprotein convertase subtilisin/kexin type 9

Declarations

Ethics approval and consent to participate

The Ethics Committee of the Third Affiliated Hospital of Zhengzhou University granted approval for the study on April 4, 2023 (2023-042-01).

Consent for publication

All authors consented to the final version submitted for publication.

Availability of data and materials

The data sets employed and analyzed in this research can be obtained upon request from the corresponding author.

Competing interest

The authors have no conflicts of interest to report.

Funding

The research received funding from Henan Provincial Science and Technology Research and Development Fund (Grant Number 182102410020).

Authors' contributions

XT and YJX conceived and designed research; YJB, JJL, LH and DL analyzed data; CCZ, YXW and PKL prepared tables; MZ, ZZS and LLH drafted manuscript; XT, CCZ, YXW and YJX edited and corrected manuscript; All co-authors participated in drafting the article.

Acknowledgments

The authors express profound gratitude to the patients and nurses who participated in and contributed to this study.

References

1. Shan Z, Wang L: **Guidelines for prevention and management of thyroid diseases during pregnancy and perinatal period**. *Chin J Endocrinol Metab* 2022, **38**(7):539-551.
2. De Leo S, Pearce EN: **Autoimmune thyroid disease during pregnancy**. *The Lancet Diabetes & Endocrinology* 2018, **6**(7):575-586.
3. Rao M, Zeng Z, Zhou F, Wang H, Liu J, Wang R, Wen Y, Yang Z, Su C, Su Z *et al*: **Effect of levothyroxine supplementation on pregnancy loss and preterm birth in women with subclinical hypothyroidism and thyroid autoimmunity: a systematic review and meta-analysis**. *Hum Reprod Update* 2019, **25**(3):344-361.
4. Zhang Y, Sun W, Zhu S, Huang Y, Huang Y, Gao Y, Zhang J, Yang H, Guo X: **The Impact of Thyroid Function and TPOAb in the First Trimester on Pregnancy Outcomes: A Retrospective Study in Peking**. *J Clin Endocrinol Metab* 2020, **105**(3).

5. Karbownik-Lewińska M, Stępniak J, Żurawska A, Lewiński A: **Less Favorable Lipid Profile and Higher Prevalence of Thyroid Antibodies in Women of Reproductive Age with High-Normal TSH—Retrospective Study**. *International Journal of Environmental Research and Public Health* 2020, **17**(6).
6. Wang L, O'Kane AM, Zhang Y, Ren J: **Maternal obesity and offspring health: Adapting metabolic changes through autophagy and mitophagy**. *Obesity Reviews* 2023, **24**(7):e13567.
7. Rohlfing AB, Nah G, Ryckman KK, Snyder BD, Kasarek D, Paynter RA, Feuer SK, Jelliffe-Pawlowski L, Parikh NI: **Maternal cardiovascular disease risk factors as predictors of preterm birth in California: a case-control study**. *BMJ open* 2020, **10**(6):e034145.
8. Chen SC, Lee CN, Hu FC, Kuo CH, Lin MW, Chen KY, Tai YY, Lin CH, Yen IW, Lin SY *et al*: **Gestational hypertriglyceridemia and adverse pregnancy outcomes: A search for cutoffs using generalized additive models**. *Diabetes res clin pr* 2022, **186**(null):109820.
9. Pimentel M, Saad RJ, Long MD, Rao SSC: **ACG Clinical Guideline: Small Intestinal Bacterial Overgrowth**. *American journal of gastroenterology* 2020, **115**(2):165-178.
10. Chen Y, Chen X, Chen P, Chen X, Pan L, Han L, Zhu T: **Alteration of the Gut Microbiota in Missed Abortion**. *Indian journal of microbiology* 2023, **63**(1):106-119.
11. Kosińska-Kaczyńska K: **Placental Syndromes-A New Paradigm in Perinatology**. *International journal of environmental research and public health* 2022, **19**(12):null.
12. Alexander EK, Pearce EN, Brent GA, Brown RS, Chen H, Dosiou C, Grobman WA, Laurberg P, Lazarus JH, Mandel SJ *et al*: **2017 Guidelines of the American Thyroid Association for the Diagnosis and Management of Thyroid Disease During Pregnancy and the Postpartum**. *Thyroid* 2017, **27**(3):315-389.
13. Rezaie A, Buresi M, Lembo A, Lin H, McCallum R, Rao S, Schmulson M, Valdovinos M, Zakko S, Pimentel M: **Hydrogen and Methane-Based Breath Testing in Gastrointestinal Disorders: The North American Consensus**. *Am j gastroenterol* 2017, **112**(5):775-784.
14. Gwee KA, Bak YT, Ghoshal UC, Gonlachanvit S, Lee OY, Fock KM, Chua AS, Lu CL, Goh KL, Kositchaiwat C *et al*: **Asian consensus on irritable bowel syndrome**. *J gastroen hepatol* 2010, **25**(7):1189-1205.
15. Lee SY, Pearce EN: **Assessment and treatment of thyroid disorders in pregnancy and the postpartum period**. *Nat rev endocrinol* 2022, **18**(3):158-171.
16. Alexander EK, Marqusee E, Lawrence J, Jarolim P, Fischer GA, Larsen PR: **Timing and magnitude of increases in levothyroxine requirements during pregnancy in women with hypothyroidism**. *New engl j med* 2004, **351**(3):241-249.
17. Zhu D: **Effect of Levothyroxine on thyroid function and maternal and fetal outcomes in pregnant women with hypothyroidism**. *Electronic Journal of Practical Gynecological Endocrinology* 2023, **10**(14):90-92.
18. Xu Y, Zhao Y, Xu X, Yan Q, Yang L: **Serum lipid profile in relation to free thyroxine and the effect of levothyroxine treatment on lipids in patients with isolated hypothyroxinemia during pregnancy: a single-center retrospective study**. *Lipids Health Dis* 2022, **21**(1):142.
19. Michalopoulou G, Alevizaki M, Piperingos G, Mitsibounas D, Mantzos E, Adamopoulos P, Koutras DA: **High serum cholesterol levels in persons with 'high-normal' TSH levels: should one extend the definition of subclinical hypothyroidism?** *European journal of endocrinology* 1998, **138**(2):141-145.
20. Sinha RA, You SH, Zhou J, Siddique MM, Bay BH, Zhu X, Privalsky ML, Cheng SY, Stevens RD, Summers SA *et al*: **Thyroid hormone stimulates hepatic lipid catabolism via activation of autophagy**. *Journal of clinical investigation* 2012, **122**(7):2428-2438.
21. Sinha RA, Singh BK, Yen PM: **Direct effects of thyroid hormones on hepatic lipid metabolism**. *Nature Reviews Endocrinology* 2018, **14**(5):259-269.

22. Sadik NA, Rashed LA, El-Sawy SS: **The Relationship of Circulating Proprotein Convertase Subtilisin/Kexin Type 9 With TSH and Lipid Profile in Newly Diagnosed Patients With Subclinical and Overt Hypothyroidism**. *Clin Med Insights Endocrinol Diabetes* 2022, **15**(null):11795514221093317.
23. Duntas LH, Brenta G: **Thyroid hormones: a potential ally to LDL-cholesterol-lowering agents**. *Horm-int j endocrino* 2016, **15**(4):500-510.
24. Komiyama M, Takahashi N, Yada Y, Koike Y, Honma Y, Aihara T, Momoi M: **Hypothyroxinemia and effectiveness of thyroxin supplementation in very low birth weight infants with abdominal distension and poor weight gain**. *Early hum dev* 2009, **85**(4):267-270.
25. Leite G, Rezaie A, Mathur R, Barlow GM, Rashid M, Hosseini A, Wang J, Parodi G, Villanueva-Millan MJ, Sanchez M *et al*: **Defining Small Intestinal Bacterial Overgrowth by Culture and High Throughput Sequencing**. *Clinical Gastroenterology and Hepatology* 2023, **null**(null):null.
26. Knezevic J, Starchl C, Tmava Berisha A, Amrein K: **Thyroid-Gut-Axis: How Does the Microbiota Influence Thyroid Function?** *Nutrients* 2020, **12**(6):null.
27. Wang B, Xu Y, Zhang M, Zhang J, Hou X, Li J, Cai Y, Sun Z, Ban Y, Wang W: **Oral and intestinal microbial features in pregnant women with hypothyroidism and their correlations with pregnancy outcomes**. *American journal of physiology-endocrinology and metabolism* 2020, **319**(6):E1044-e1052.
28. Lasa M, Contreras-Jurado C: **Thyroid hormones act as modulators of inflammation through their nuclear receptors**. *Frontiers in endocrinology* 2022, **13**(null):937099.
29. Spalinger MR, Sayoc-Becerra A, Santos AN, Shawki A, Canale V, Krishnan M, Niechcial A, Obialo N, Scharl M, Li J *et al*: **PTPN2 Regulates Interactions Between Macrophages and Intestinal Epithelial Cells to Promote Intestinal Barrier Function**. *Gastroenterology* 2020, **159**(5):1763-1777.e1714.
30. Hao Y, Xu Y, Ban Y, Li J, Wu B, Ouyang Q, Sun Z, Zhang M, Cai Y, Wang M *et al*: **Efficacy evaluation of probiotics combined with prebiotics in patients with clinical hypothyroidism complicated with small intestinal bacterial overgrowth during the second trimester of pregnancy**. *Frontiers in cellular and infection microbiology* 2022, **12**(null):983027.
31. Yang Y, Xiao L: **Investigation on the effect of Levothyroxine treatment on the adverse pregnancy outcome in euthyroid pregnant women with TPOAb positive**. *J of Pub Health and Prev Med* 2018, **29**(02):70-73.
32. Di Girolamo R, Liberati M, Silvi C, D'Antonio F: **Levothyroxine Supplementation in Euthyroid Pregnant Women With Positive Autoantibodies: A Systematic Review and Meta-Analysis**. *Front Endocrinol (Lausanne)* 2022, **13**:759064.
33. Reilly NA, Lutgens E, Kuiper J, Heijmans BT, Wouter Jukema J: **Effects of fatty acids on T cell function: role in atherosclerosis**. *Nature Reviews Cardiology* 2021, **18**(12):824-837.
34. Macpherson ME, Skarpengland T, Hov JR, Ranheim T, Vestad B, Dahl TB, Fraz MSA, Michelsen AE, Holven KB, Fevang B *et al*: **Increased Plasma Levels of Triglyceride-Enriched Lipoproteins Associate with Systemic Inflammation, Lipopolysaccharides, and Gut Dysbiosis in Common Variable Immunodeficiency**. *Journal of clinical immunology* 2023, **43**(6):1229-1240.
35. Zheng D, Liao H, Chen S, Liu X, Mao C, Zhang C, Meng M, Wang Z, Wang Y, Jiang Q *et al*: **Elevated Levels of Circulating Biomarkers Related to Leaky Gut Syndrome and Bacterial Translocation Are Associated With Graves' Disease**. *Frontiers in endocrinology* 2021, **12**(null):796212.
36. Min Y, Wang X, Chen H, Yin G: **The exploration of Hashimoto's Thyroiditis related miscarriage for better treatment modalities**. *Int J Med Sci* 2020, **17**(16):2402-2415.
37. Adu-Gyamfi EA, Wang YX, Ding YB: **The interplay between thyroid hormones and the placenta: a comprehensive review†**. *Biol reprod* 2020, **102**(1):8-17.

38. Savasan ZA, Romero R, Chaiworapongsa T, Kusanovic JP, Kim SK, Mazaki-Tovi S, Vaisbuch E, Mittal P, Ogge G, Madan I *et al*: **Evidence in support of a role for anti-angiogenic factors in preterm prelabor rupture of membranes**. *Journal of Maternal-Fetal & Neonatal Medicine* 2010, **23**(8):828-841.
39. Kianpour M, Aminorroaya A, Amini M, Feizi A, Aminorroaya Yamini S, Janghorbani M: **Thyroid-stimulating hormone (TSH) serum levels and risk of spontaneous abortion: A prospective population-based cohort study**. *Clinical endocrinology* 2019, **91**(1):163-169.
40. Kakita-Kobayashi M, Murata H, Nishigaki A, Hashimoto Y, Komiya S, Tsubokura H, Kido T, Kida N, Tsuzuki-Nakao T, Matsuo Y *et al*: **Thyroid Hormone Facilitates in vitro Decidualization of Human Endometrial Stromal Cells via Thyroid Hormone Receptors**. *Endocrinology* 2020, **161**(6):null.
41. Nazarpour S, Ramezani Tehrani F, Simbar M, Tohidi M, Alavi Majd H, Azizi F: **Effects of levothyroxine treatment on pregnancy outcomes in pregnant women with autoimmune thyroid disease**. *Eur j endocrinol* 2017, **176**(2):253-265.
42. Huget-Penner S, Feig DS: **Maternal thyroid disease and its effects on the fetus and perinatal outcomes**. *Prenatal diag* 2020, **40**(9):1077-1084.
43. Mayhew CE, Simonson KR, Ellsworth Bowers ER: **Antepartum Care for Pregnant People with Overt Hypothyroidism, Subclinical Hypothyroidism, and Positive Thyroid Autoantibodies**. *J midwifery wom heal* 2022, **67**(3):295-304.

9% Overall Similarity

Top sources found in the following databases:

- 8% Internet database
- 7% Publications database
- Crossref database
- Crossref Posted Content database

TOP SOURCES

The sources with the highest number of matches within the submission. Overlapping sources will not be displayed.

#	Source	Type	Similarity
1	researchsquare.com	Internet	<1%
2	jamanetwork.com	Internet	<1%
3	insights.ovid.com	Internet	<1%
4	mdpi.com	Internet	<1%
5	Yan-Xin Jiang, Wei-Jie Sun, Yang Zhang, Yu Huang et al. "Thyroid funct...	Crossref	<1%
6	medrxiv.org	Internet	<1%
7	assets.researchsquare.com	Internet	<1%
8	hindawi.com	Internet	<1%
9	longdom.org	Internet	<1%

10	link.springer.com<br>Internet	<1%
11	cardiothoracicsurgery.biomedcentral.com<br>Internet	<1%
12	scialert.net<br>Internet	<1%
13	dovepress.com<br>Internet	<1%
14	frontiersin.org<br>Internet	<1%
15	pubmed.ncbi.nlm.nih.gov<br>Internet	<1%
16	Park, H.T.. "Thyroid stimulating hormone is associated with metabolic ...<br>Crossref	<1%
17	"Thyroid Diseases", Springer Science and Business Media LLC, 2018<br>Crossref	<1%
18	Yang Zhang, Weijie Sun, Sainan Zhu, Youyuan Huang, Yu Huang, Ying G...<br>Crossref	<1%
19	archrazi.areeo.ac.ir<br>Internet	<1%
20	"Meeting Abstracts from the 2019 IFFS Shanghai World Congress", Glo...<br>Crossref	<1%
21	"Thyroid and Heart", Springer Science and Business Media LLC, 2020<br>Crossref	<1%

22	ijpras.com<br>Internet	<1%
23	elsevier.es<br>Internet	<1%
24	e-century.us<br>Internet	<1%
25	researchgate.net<br>Internet	<1%
26	Feifei Shao, Rui Li, Qian Guo, Rui Qin, Wenxiu Su, Huiyong Yin, Limin Ti...<br>Crossref	<1%
27	Flora Veltri, Pierre Kleynen, Lidia Grabczan, Alexandra Salajan, Serge R...<br>Crossref	<1%
28	Jingjing Li, Yajuan Xu, Zongzong Sun, Yanjun Cai et al. "Differential lipi...<br>Crossref	<1%
29	bmcpregnancychildbirth.biomedcentral.com<br>Internet	<1%
30	c.coek.info<br>Internet	<1%
31	ia902804.us.archive.org<br>Internet	<1%
32	Bo Wu, Yajuan Xu, Yanjie Ban, Miao Zhang et al. "Correlation between t...<br>Crossref	<1%
33	Ning Yuan, Jianbin Sun, Xin Zhao, Jing Du, Min Nan, Qiaoling Zhang, Xi...<br>Crossref	<1%

34	Zheng Yang, Haoyu Wang, Yifu Liu, Yan Feng, Yang Xiang, Jing Li, Zhon...	Crossref	<1%
35	bsj.uobaghdad.edu.iq	Internet	<1%
36	doaj.org	Internet	<1%
37	ec.bioscientifica.com	Internet	<1%
38	es.scribd.com	Internet	<1%
39	old.edouniversity.edu.ng	Internet	<1%
40	worldwidescience.org	Internet	<1%
41	jove.com	Internet	<1%
42	thieme-connect.com	Internet	<1%
43	"Advanced Practice in Endocrinology Nursing", Springer Science and B...	Crossref	<1%
44	ncbi.nlm.nih.gov	Internet	<1%

Excluded from Similarity Report

- Bibliographic material
- Manually excluded text blocks

EXCLUDED TEXT BLOCKS

Savasan ZA, Romero R, Chaiworapongsa T, Kusanovic JP, Kim SK, Mazaki-Tovi S, ...
Emmanuel Bujold, Stéphanie Roberge, Sylvie Tapp, Yves Giguère. "Opinion & HypothesisCould early aspirin p...

Kianpour M, Aminorroaya A, Amini M, Feizi A, Aminorroaya Yamini S, Janghorbani ...
nmrc.mui.ac.ir

Kakita-Kobayashi M, Murata H, Nishigaki A, Hashimoto Y, Komiya S, Tsubokura H, ...
www.mdpi.com

Nazarpour S, Ramezani Tehrani F, Simbar M, Tohidi M, Alavi Majd H, Azizi F: Effect...
jamanetwork.com

Huget-Penner S, Feig DS: Maternal thyroid disease and its effects on the fetus and...
pubmed.ncbi.nlm.nih.gov

Mayhew CE, Simonson KR, Ellsworth Bowers ER: Antepartum Care for Pregnant Pe...
wjgnet.com

Journal of Maternal
www2.med.wayne.edu

1 Yajuan Xu,1* Yanjie Ban,1 Jingjing Li,1
link.springer.com
